# Gray Matter Atrophy Is Associated With Cognitive Impairment in Patients With Presbycusis: A Comprehensive Morphometric Study

**DOI:** 10.3389/fnins.2018.00744

**Published:** 2018-10-23

**Authors:** Fuxin Ren, Wen Ma, Muwei Li, Huaiqiang Sun, Qian Xin, Wei Zong, Weibo Chen, Guangbin Wang, Fei Gao, Bin Zhao

**Affiliations:** ^1^Shandong Medical Imaging Research Institute, Shandong University, Jinan, China; ^2^Department of Otolaryngology, Jinan Central Hospital, Shandong University, Jinan, China; ^3^Vanderbilt University Institute of Imaging Science, Vanderbilt University, Nashville, TN, United States; ^4^Huaxi MR Research Center, Department of Radiology, West China Hospital of Sichuan University, Chengdu, China; ^5^Central Laboratory, The Second Hospital of Shandong University, Jinan, China; ^6^Philips Healthcare, Shanghai, China

**Keywords:** presbycusis, cognitive impairment, hearing loss, GM atrophy, surface-based morphometry

## Abstract

Presbycusis (PC) is characterized by bilateral sensorineural hearing loss at high frequencies and speech-perception difficulties in noisy environments and has a strikingly detrimental impact on cognitive function. As the neural consequences of PC may involve the whole brain, we hypothesized that patients with PC would show structural alterations not only in the auditory cortex but also in the cortexes involved in cognitive function. The purpose of this study was to use surface-based morphometry (SBM) analysis to elucidate whole-brain structural differences between patients with PC and age-matched normal hearing controls. Three-dimensional T1-weighted MR images of 26 patients with mild PC and 26 age-, sex- and education-matched healthy controls (HCs) were acquired. All participants underwent a battery of neuropsychological tests. Our results revealed gray matter atrophy in several auditory cortical areas, nodes of the default mode network (DMN), including the bilateral precuneus and inferior parietal lobule, the right posterior cingulate cortex (PCC), and the right insula of patients with PC compared to that in the HCs. Our findings also revealed that hearing loss was associated with reduced gray matter volume in the right primary auditory cortex of patients with PC. Moreover, structural alterations in the nodes of the DMN were associated with cognitive impairments in PC patients. Additionally, this study provides evidence that a thicker right insula is associated with better speech perception in patients with PC. Based on these findings, we argue that the onset of PC seems to trigger its own cascade of conditions, including a need for increased cognitive resources during speech comprehension, which might lead to auditory and cognition-related cortical reorganization.

## Introduction

Age-related hearing loss, also known as presbycusis (PC), is characterized by bilateral sensorineural hearing loss at high frequencies, slowed central processing of acoustic information and speech-perception difficulties in noisy environments (Gates and Mills, 2005). PC is the most common sensory deficit in the aging population and is associated with a diminished quality of life. Recently,many large population-based longitudinal studies have suggested that age-related hearing loss is independently associated with cognitive decline and that patients with PC are more likely to develop dementia (Lin et al., 2011a,b; Gurgel et al., 2014). For instance, one cross-sectional study found that patients with PC showed worse performance than normal hearing controls on the Digit Symbol Substitution Test, which evaluates psychomotor speed and executive function (Lin, 2011). Moreover, compared with normal hearing controls, PC patients with mild, moderate and severe hearing loss are, respectively, two, three and five times as likely to develop dementia (Lin et al., 2011a). In line with these findings, researchers have realized that PC may have a strikingly detrimental impact on cognitive function (Mudar and Husain, 2016; Hewitt, 2017; Jayakody et al., 2018). Given that hearing loss is relatively easier to remediate than other risk factors for dementia and Alzheimer’s disease (Mudar and Husain, 2016), it is highly important to illuminate the neural mechanisms that underlie PC-related cognitive decline.

Magnetic resonance imaging (MRI) has become a novel and widely used technique to investigate the pathogenesis of various neuropsychiatric disorders. In one structural MRI study, patients with PC exhibited decreases in gray matter (GM) volume and thickness in the bilateral auditory cortexes compared with normal hearing young adults (Profant et al., 2014). Other studies have reported decreases in GM volume in the right primary auditory cortex that correlated with poorer hearing ability in older adults (Peelle et al., 2011). In one functional magnetic resonance imaging (fMRI) study, compared with normal hearing young adults, patients with PC showed higher blood-oxygen-level-dependent activation responses to acoustical stimuli in the temporal lobes (Ouda et al., 2015). However, these studies lacked age-matched normal hearing controls; thus, the further validation of these findings is required. In our previous studies using proton magnetic resonance spectroscopy, compared with age-matched normal hearing controls, decreased concentrations of gamma-aminobutyric acid (GABA), the main inhibitory neurotransmitter in the central auditory system, have been found in the bilateral auditory cortexes in patients with PC (Gao et al., 2015). However, the aforementioned studies and our studies have mainly focused on structural, functional and metabolic changes in the auditory cortex.

The strong connection between PC and cognitive decline has been explained by several hypotheses (Humes et al., 2013; Wayne and Johnsrude, 2015). Several researchers have suggested that even relatively mild levels of hearing loss can lead to increased listening effort, including the need for increased cognitive resources to understand acoustically degraded speech, and that reduced hearing ability has cascading consequences for the neural processes supporting both speech perception and cognition (Wingfield and Peelle, 2012; Peelle and Wingfield, 2016). As the neural consequences of PC may involve the whole brain, we should also consider neural alterations in nonauditory cortical regions. Therefore, the purpose of this study was to use surface-based morphometric (SBM) analysis to elucidate structural differences in the whole brain between patients with PC and age-matched normal hearing controls. Furthermore, the relationships between structural changes and cognitive decline in patients with PC were analyzed. We hypothesized that patients with PC would present structural alterations not only in the auditory cortex but also in the cortical regions involved in cognitive functions.

## Materials and Methods

### Participants

The study was approved by the Shandong University institutional review board and each participant provided informed consent. Twenty-six patients with mild PC (PC group, 14 males/12 females, mean age, 64.38 ± 3.24 years) visiting the Department of Otolaryngology at our local hospital were recruited for this study (Table 1). Hearing loss was assessed by the speech-frequency pure tone average (PTA) of thresholds at 0.5, 1, 2, and 4 kHz (air conduction) in the better hearing ear as per the definition of hearing loss adjudicated by the World World Health Organization [WHO], 1991. The PTA value of 25 decibels hearing level (dB HL) was accepted as the normal hearing threshold limit (Lin et al., 2011b). Inclusion criteria were the following: (1) hearing loss: PTA> 25 dB HL in the better hearing ear; (2) age≥ 60 years. Exclusion criteria were the following: (1) ear diseases that affected hearing thresholds and sensorineural hearing losses other than PC; (2) previous history of otologic surgery, ototoxic drug therapy, noise exposure, or hearing aid use; (3) asymmetric hearing loss with a difference in air conduction thresholds exceeding 20 dB at least two frequencies between 0.5, 1, 2, and 4 kHz; (4) conductive hearing loss (a mean air-bone difference at 0.5, 1, 2, and 4 kHz) > 10 dB in one or both ears; and (5) tinnitus, head trauma, lesions of the facial nerve, disorders of the cervical spine, or neurological or psychiatric diseases.

**Table 1:** Participants’ demographic and clinical data.

Characteristics	PC (*n* = 26)	HCs (*n* = 26)	*p*-value
Gender (male/female)	14/12	13/13	0.781
Age (years)	64.38 3.24	64.96 ± 3.00	0.508
Education (years)	11.31 ± 2.17	11.15 ± 2.68	0.821
Disease duration (years)	5.23 ± 2.08	-	-
PTA	32.65 ± 5.34	14.69 ± 3.86	<0.001^∗^
SRT	32.63 ± 4.31	15.90 ± 4.49	<0.001^∗^
MMSE	27.35 ± 0.98	27.73 ± 0.83	0.132
MoCA	25.77 ± 1.11	26.19 ± 0.69	0.105
Anxiety	3.92 ± 1.57	3.46 ± 2.04	0.366
Depression	3.73 ± 1.76	3.38 ± 1.83	0.490
AVLT	57.08 ± 4.51	59.88 ± 8.53	0.144
Stroop	135.88 ± 11.18	125.69 ± 11.10	0.002^∗^
SDMT	42.31 ± 9.30	51.31 ± 13.07	0.006^∗^
TMT-A	42.08 ± 5.84	38.15 ± 8.88	0.066
TMT-B	106.58 ± 13.34	96.08 ± 19.05	0.026^∗^


*The data are presented as means ± standard deviations. ^∗^Indicates *p* < 0.05. PC, presbycusis; HCs, healthy controls; PTA, pure tone average; SRT, speech reception threshold; MMSE, Mini Mental State Examination; MoCA, Montreal Cognitive Assessment; AVLT, Auditory Verbal Learning Test; SDMT, Symbol Digit Modalities Test; TMT, Trail-Making Test; levels of anxiety and depression were assessed according to the Hospital Anxiety and Depression Scale (HADS).*

Twenty-six age-, sex- and education-level matched healthy controls (HCs; control group, 13 males/13 females, mean age, 64.96 ± 3.0 years; PTA ≤ 25 dB HL in the better hearing ear) were recruited for this study (Table 1). All controls were in good health and had no history of neurological or psychiatric diseases. All participants were right-handed, as determined by the Li’s handedness inventory (Gong et al., 2005; Hatta, 2007).

### Assessment of Auditory Function

An otoscopic examination was performed for all participants to remove cerumen and confirm the presence of an intact tympanic membrane. The auditory function of all participants was evaluated using tympanometry and pure tone audiometry. Tympanometry was performed with a GSI Tympstar to confirm optimal middle ear conditions. Pure tone audiometry was performed with a GSI AudioStar Pro audiometer coupled with TDH-50P Telephonics headphones. Bone conduction thresholds were measured at 0.25, 0.5, 1, 2, and 4 kHz, and air conduction thresholds were measured at 0.125, 0.25, 0.5, 1, 2, 4, and 8 kHz. Hearing thresholds were detected with a resolution of 5 dB steps. The PTA values for all participants’ ears were calculated.

Speech reception threshold (SRT) was measured in quiet conditions. SRT testing was conducted using the automated HOPE software for the presentation and scoring of spondee words. The SRT testing in this software is conducted according to the American Speech-language Hearing Association recommended SRT testing guidelines. The process is as follows: first, an initial sound intensity is determined based on the PTA hearing threshold where five spondee words are correctly identified. If these words are not identified correctly, the software will prompt “Increase initial sound intensity”. Then, the software will automatically control the playback intensity steps: 5 dB decreases for every five words played. When the patient fails to recognize the five words at certain intensity, the test is terminated. The software counts the number of words that the patient successfully recognized during the entire step-down process and subtracts this from the initial intensity, plus a 2.5 dB correction factor, which is the patient’s SRT.

### Assessment of Cognitive Function

The participants’ cognitive status were tested using the Mini Mental State Examination (MMSE) and Montreal Cognitive Assessment (MoCA) for general cognitive function (Galea and Woodward, 2005; Nasreddine et al., 2005), the Auditory Verbal Learning Test (AVLT, Chinese version) for verbal learning and memory (Zhao et al., 2012), the Stroop color word interference test for attention (Savitz and Jansen, 2003), the Symbol Digit Modalities Test (SDMT) for psychomotor speed (Van Schependom et al., 2014), and the Trail-Making Test for executive control (Sanchez-Cubillo et al., 2009). Levels of anxiety and depression were assessed according to the Hospital Anxiety and Depression Scale (HADS) (Zigmond and Snaith, 1983). Each participant took approximately 60 min to complete all tests, which were completed in a fixed order.

### MRI Acquisition

All participants were scanned on a 3T scanner (Philips Achieva TX, Best, Netherlands) using an eight-channel phased-array head coil as a receiver. T1-weighted three-dimensional TFE images were used as a localizer and acquired with the following parameters: TR = 8.1 ms; TE = 3.7 ms; slice thickness = 1 mm; field of view = 24 cm × 24 cm; and flip angle = 8°. Images were reconstructed with 1 mm × 1 mm × 1 mm isotropic voxels.

### Surface-Based Morphometric Analysis

All the T1-weighted images were processed with surface stream and volume stream that were integrated in FreeSurfer software (version 5.3^1^). The surface stream constructs the boundary models of white matter, gray matter as well as the pial surface, from which, the anatomical measurements, such as cortical thickness and curvature, could be obtained at each point on the cortex. Then the cortex was segmented into 74 regions of interest (ROIs) using the volume stream, which consists three major steps. First, T1 image is spatially aligned to a standard coordinate, namely, Montreal Neurological Institute (MNI) space, using affine registration which is insensitive to local anatomical difference and used to maximize the accuracy of final segmentation. This is followed by a refined high-dimensional nonlinear registration, which warps the T1 image to an MNI-space atlas whose cortical area has been manually segmented to 74 ROIs. The non-linear registration allows for the point-to-point correspondence between T1 image and the atlas, and therefore enable the automatic segmentation of the T1 image. Consequently, the normalized volume [dividing by intracranial volume (ICV)], average thickness and average curvature within each ROI could be obtained for accurate characterization of local anatomical morphometry of cortical areas. The Shapiro-Wilk normality test was used to test for normality of distribution. For each ROI, group differences in these measurements between the PC and HCs groups were assessed by two-sample *t*-test (Gutiérrez-Galve et al., 2010; Vainik et al., 2018). The age, sex and education level were modeled as covariates. The analysis was corrected for multiple comparisons using the FDR criterion and the statistical significance was set at *p* < 0.05.

### Statistical Analysis

We used the two-tailed *t*-test to assess group differences in demographic and clinical characteristics. Gender-specific group differences were analyzed using the chi-square test. *P*-values of less than 0.05 were accepted as significant. Partial correlation analyses were performed to explore the potential relationships between structural changes and cognitive impairments or audiological outcomes in the PC group (controlled for age, sex, education level). All statistical analyses were conducted using PASW software (version 17.0, Chicago, IL, United States).

## Results

### Demographic and Clinical Characteristics

**FIGURE 1:**
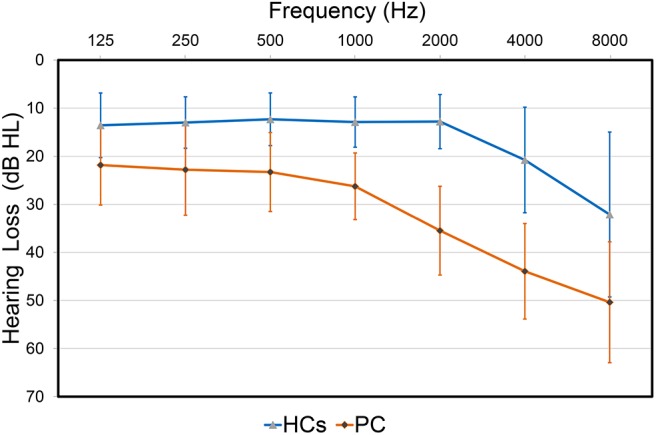
Hearing thresholds of the presbycusis (PC) and healthy controls (HCs) groups (means ± standard deviation) in air conduction. Hearing thresholds from both ears are averaged.

The demographic and clinical characteristics are listed in Table 1. The two groups did not exhibit significant differences in age, gender or education level. Compared to the HCs, patients with PC performed worse on the Stroop, SDMT and TMT-B tests (*p* < 0.05) (Table 1). All participants had a type A curve (normal middle ear function) on tympanometry. There was no significant difference in PTA or SRT between the left and right ears in the PC and HCs groups, thus the thresholds of both ears were averaged in each group. Average hearing thresholds of the PC and HCs groups were shown in Figure 1. The PTA and SRT were significantly higher in patients with PC than in HCs (PTA, *p* < 0.001; SRT, *p*<0.001) (Table 1).

### Group Differences in Cortical Morphology

**FIGURE 2:**
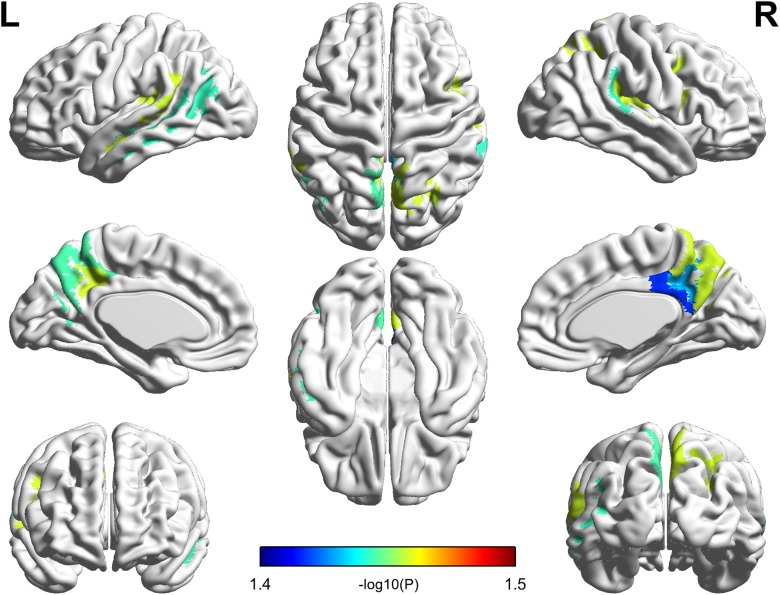
Compared with healthy controls, patients with presbycusis showed decreased volume in the left superior temporal sulcus; the right posterior cingulate cortex and transverse temporal sulcus; and the bilateral precuneus, temporal plane of the superior temporal gyrus and subparietal sulcus (*p* < 0.05, false discovery rate corrected).

All cortical variables were normal distribution (*p* > 0.05) except the cortex thickness of the right insular of PC group. The quantile–quantile plot (Wilk and Gnanadesikan, 1968) was used to check for major deviations from the normal distribution, then one patient’s data was excluded from the cortex thickness of the right insular. The significant parcel-wise differences between the PC and HCs groups in cortical volume are illustrated in Figure 2. Compared with the HCs group, the PC group showed decreased volume in the left superior temporal sulcus; the right posterior cingulate cortex (PCC) and transverse temporal sulcus; and the bilateral precuneus, temporal plane of the superior temporal gyrus and subparietal sulcus.

**FIGURE 3:**
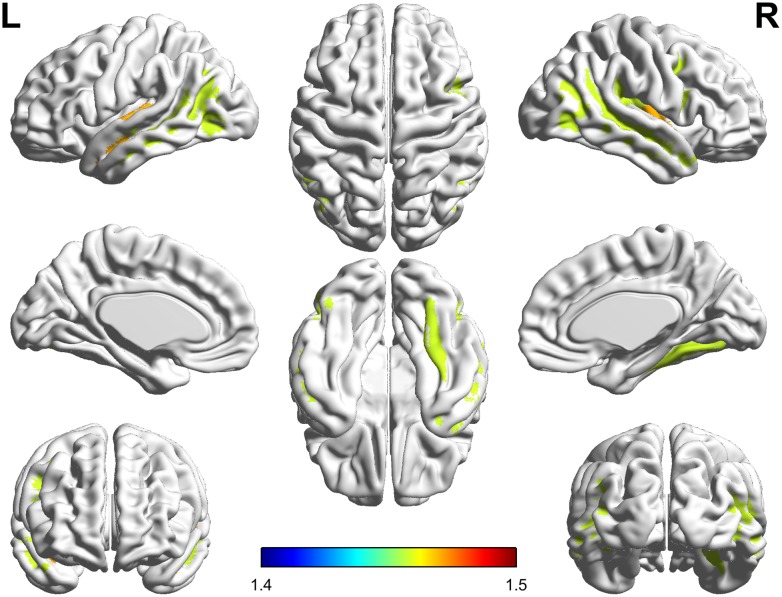
Compared with healthy controls, patients with presbycusis showed reduced thickness in the left Heschl’s gyrus; the right insular gyrus and planum polare of the superior temporal gyrus; and bilateral superior temporal sulcus (*p* < 0.05, false discovery rate corrected).

The significant parcel-wise differences between the PC and HCs groups in cortical thickness are illustrated in Figure 3. Compared with the HCs group, the PC group showed reduced thickness in the left Heschl’s gyrus (HG); the right insular gyrus and planum polare of the superior temporal gyrus; and bilateral superior temporal sulcus. There were no significant parcel-wise differences between the PC and HCs groups in cortical curvature.

### Correlations Between Clinical Characteristics and Cortical Morphology

**FIGURE 4:**
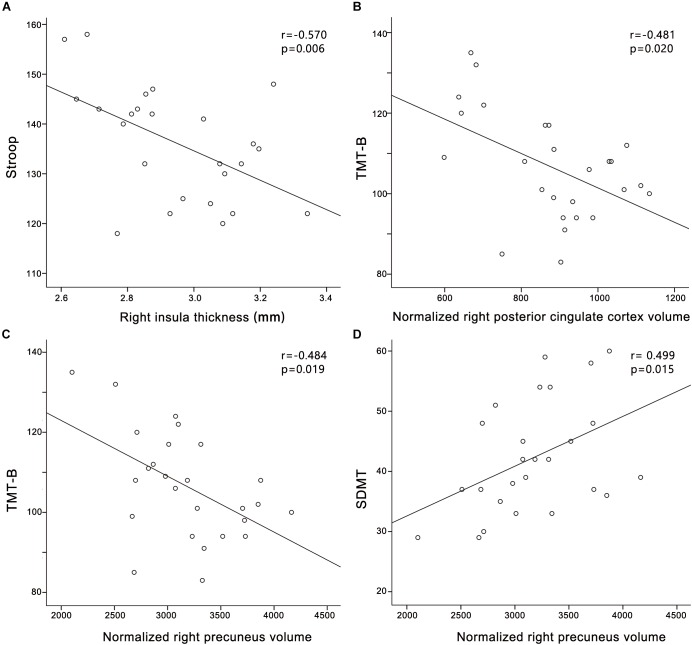
Correlations between structural changes and cognitive impairments in the presbycusis group. **(A)** Stroop scores were negatively correlated with the cortical thickness of the right insula (*r* = –0.570, *p* = 0.006); **(B)** TMT-B scores were negatively correlated with the cortical volume of the right posterior cingulate cortex (*r* = –0.481, *p* = 0.020); **(C)** TMT-B scores were negatively correlated with the cortical volume of the right precuneus (*r* = –0.484, *p* = 0.019); **(D)** SDMT scores were positively associated with the cortical volume of the right precuneus (*r* = 0.499, *p* = 0.015). Normalized cortical volume = (cortical volume/intracranial volume) ^∗^10^6^.

In the PC group (Figure 4), partial correlation analyses revealed that Stroop scores were negatively correlated with the cortical thickness of the right insula (*r* = -0.570, *p* = 0.006); TMT-B scores were negatively correlated with the cortical volume of the right PCC (*r* = -0.481, *p* = 0.020); TMT-B scores were negatively correlated with the cortical volume of the right precuneus (*r* = -0.484, *p* = 0.019); SDMT scores were positively associated with the cortical volume of the right precuneus (*r* = 0.499, *p* = 0.015). In the HCs group (Supplementary Figure S1), no correlations were observed between cognitive scores and cortical morphology. In all participants (Supplementary Figure S3), Stroop scores were negatively correlated with the cortical thickness of the right insula (*r* = -0.394, *p* = 0.006).

**FIGURE 5:**
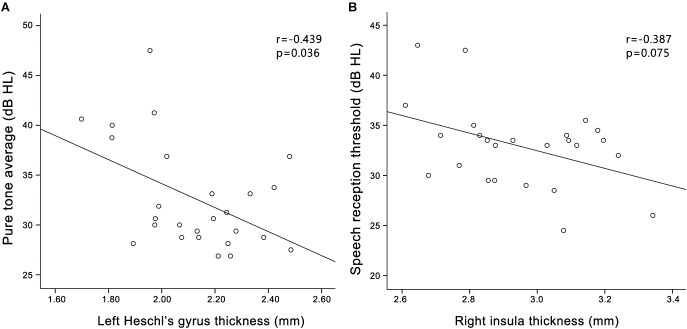
Correlations between structural changes and audiological outcomes in the presbycusis group. **(A)** PTA was negatively correlated with the cortical thickness of the left HG (*r* = –0.439, *p* = 0.036); **(B)** A trend toward correlation was seen between SRT and the cortical thickness of the right insula (*r* = –0.387, *p* = 0.075).

In the PC group (Figure 5), partial correlation analyses revealed that PTA was negatively correlated with the cortical thickness of the left HG (*r* = -0.439, *p* = 0.036), and a trend toward correlation was seen between SRT and the cortical thickness of the right insula (*r* = -0.387, *p* = 0.075). In the HCs group (Supplementary Figure S2), PTA was negatively correlated with the cortical thickness of the left HG (*r* = -0.460, *p* = 0.027). In all participants (Supplementary Figure S4), PTA was negatively correlated with the cortical thickness of the left HG (*r* = -0.556, *p* < 0.001); SRT was negatively correlated with the cortical thickness of the right insula (*r* = -0.552, *p* < 0.001).

## Discussion

Our study demonstrated GM atrophy in several auditory cortical areas; nodes of the default mode network (DMN), including the bilateral precuneus, inferior parietal lobule and right PCC; and the right insula of patients with PC compared to those of age- and sex-matched normal hearing controls. Our findings also revealed that hearing loss was associated with reduced cortical thickness in the left HG of patients with PC. Moreover, structural alterations in nodes of the DMN were associated with cognitive impairments in PC patients. Additionally, a trend toward correlation was demonstrated between SRT and the cortical thickness of the right insula in patients with PC. To the best of our knowledge, this is the first study to systematically elucidate structural differences in the whole brain of patients with PC and their correlations with cognitive impairments.

The plastic reorganization of sensory cortexes often occurs when sensory input is degraded. In our study, decreased cortical volume and/or thickness in patients with PC were observed in several auditory cortical areas, such as the left HG, the right planum polare of the superior temporal gyrus and bilateral temporal plane of the superior temporal gyrus. These findings could demonstrate corresponding brain dysfunctions that are related to long-term hearing loss. To our knowledge, only one study has reported structural changes in the GM of patients with PC. In that study, the GM volume and thickness of bilateral HG and the planum temporale were decreased in patients with mild PC in comparison with young control subjects (Profant et al., 2014). Therefore, alterations in auditory cortical areas may be mainly caused by aging rather than hearing loss. Intriguingly, our findings revealed that hearing loss was associated with reduced cortical thickness in the left HG of patients with PC, which suggests a link between hearing ability and the structural integrity of the auditory cortex. These results are consistent with previous studies indicating cortical reorganization when peripheral auditory acuity is moderately decreased in older adults (Peelle et al., 2011). HG is a crucial brain structure as it contains the primary auditory cortex, which plays an important role in auditory information processing (Langers et al., 2007; Wong et al., 2008). For patients with PC, long-term hearing loss may impair the ability of the primary auditory cortex to respond to auditory signals. Significant correlations were also observed between the cortical morphology and hearing thresholds both in the HCs group and all participants. In our study, hearing loss was assessed by the speech-frequency PTA of thresholds at 0.5, 1, 2, and 4 kHz in the better hearing ear. However, healthy controls had high frequency hearing losses, especially in 8 kHz, which were not been fully evaluated by speech-frequency PTA. Moreover, the relationships between the cortical morphology and hearing thresholds in all participants were more robust than those in PC or HCs group. One possible reason for the result could be an increase in the sample size.

Previous studies on PC have indicated that hearing loss contributes to cognitive decline (Lin, 2011; Lin et al., 2011a,b; Gurgel et al., 2014). In our study, patients with PC showed poorer performances than the HCs in most of the cognitive tests, such as the Stroop, SDMT and TMT-B tests, which indicates that patients exhibited attention, information processing speed and executive function dysfunctions. These results may possibly explain the finding of morphological alterations that involved not only the auditory cortex but also cognitive areas. The DMN is a large-scale brain network of interacting brain regions that shows increased activity at rest and plays a critical role in modulating consciousness (Raichle et al., 2001; Mantini et al., 2007). In this study, several nodes in the DMN of PC patients showed decreased cortical volume and/or thickness. These areas included the right PCC, which has been found to be associated with attention and cognitive control (Leech et al., 2011; Leech and Sharp, 2014); the bilateral precuneus, which is responsible for episodic memory and visuospatial processing (Cavanna and Trimble, 2006); and the bilateral inferior parietal lobules, which are involved in maintaining attentive control and interpretation of sensory information (Singh-Curry and Husain, 2009). The complexity of an acoustic signal such as speech requires the involvement of nonauditory cortexes for correct processing (Ouda et al., 2015). PC-related hearing loss increases listening effort, including a need for increased cognitive resources to understand acoustically degraded speech, especially in noisy places (Peelle and Wingfield, 2016). Therefore, our results presumably indicate a plastic reorganization of neural systems in patients with PC in which some nodes of the DMN become overused to compensate for the lost ability to inhibit irrelevant stimuli such as background noise. The chronic overuse of nodes of the DMN in patients with PC may eventually lead to structural modifications in these nodes, as seen in our study. Moreover, our analysis showed that impaired cognitive function scores were related to decreased cortical volume in the right precuneus and PCC. Structural alterations in the nodes of the DMN in patients with PC were associated with cognitive decline, suggesting that brain morphometry might be a potential imaging marker for PC-associated cognitive impairments.

We also found significant cortical thickness decreases in the right insula in patients with PC. Moreover, a trend toward correlation was demonstrated between SRT and the cortical thickness of the right insula in PC patients, indicating a thicker right insula is associated with better speech perception. These findings suggest that in addition to peripheral structures and the auditory cortex, cognitive areas also contribute to the ability to perceive speech in patients with PC. Virtually all high-level auditory information processes rely on the control and conditioning of cognitive processes (Cardin, 2016). The insula, a key node in cognitive control network (CCN), participates in several key auditory processes, such as auditory attention allocation and focusing on novel auditory stimuli, temporal processing, phonological processing and visual-auditory information integration (Bamiou et al., 2003; Cole and Schneider, 2007). Thus, our findings may suggest that a remodeling in cognitive control areas is necessary for auditory processing in PC patients. Previous fMRI studies of patients with USNHL have shown increased regional homogeneity (ReHo) in the right anterior insula, and functional connectivity analyses showed an enhanced relationship between the right insula and several key nodes of the DMN (Wang et al., 2014). Additionally, stroop scores were negatively correlated with the cortical thickness of the right insula in this study. Taken together with the current data showing the altered integrity of GM in key nodes of the CCN and DMN, these results indicate a functional reorganization both within and between cognitive networks in patients with PC; these findings encourage further investigations using resting-state fMRI.

There are some limitations to our study design. First, as it was a cross-sectional study, although relationships between structural changes and cognitive decline were found in patients with PC, it is still not clear whether improving hearing ability through the use of hearing aids might help preserve cognitive function. Second, there is a lack of evaluation of how the freesurfer measurements would change when using a different atlas for registration. Morphological change of elderly population, for example, larger ventricle and atrophied cortical areas, might reduce the registration accuracy when using a universal atlas. Therefore, an elderly population-specific atlas is highly expected to yield unbiased measurement and further increase the statistic power. Third, our sample size is small, and the results require confirmation in larger samples. Fourth, a more comprehensive neuropsychological battery including measures of visual memory, semantic memory, as well as additional executive processes (e.g., verbal fluency, working memory and concept formation/problem solving) is another limitation of the study.

## Conclusion

Our findings indicate that GM atrophy of key nodes in the DMN may be imaging markers for PC-related cognitive decline. Moreover, this study provides evidence that a thicker right insula is associated with better speech perception in patients with PC. Based on these findings, we argue that the onset of PC appears to trigger its own cascade of conditions, including the need for increased cognitive resources for speech comprehension, which might lead to auditory and cognition-related cortical reorganization.

## Ethics Statement

This study was carried out in accordance with the recommendations of Shandong University institutional review board with written informed consent from all subjects. All subjects gave written informed consent in accordance with the Declaration of Helsinki. The protocol was approved by the Shandong University institutional review board.

## Author Contributions

FG and BZ designed the experiments. FR, WM, and QX carried out the experiments. WZ and WC analyzed experimental results. ML and HS analyzed MRI data and developed analysis tools. GW assisted with. FR and FG wrote the manuscript.

## Conflict of Interest Statement

The authors declare that the research was conducted in the absence of any commercial or financial relationships that could be construed as a potential conflict of interest.

## Footnotes

1 https://surfer.nmr.mgh.harvard.edu
