# Supplementary figures and images for: Gray Matter Atrophy Is Associated With Cognitive Impairment in Patients With Presbycusis: A Comprehensive Morphometric Study
Source: Front Neurosci. 2018 Oct 23;12:744. doi: 10.3389/fnins.2018.00744 (PMC6205975; doi:10.3389/fnins.2018.00744)

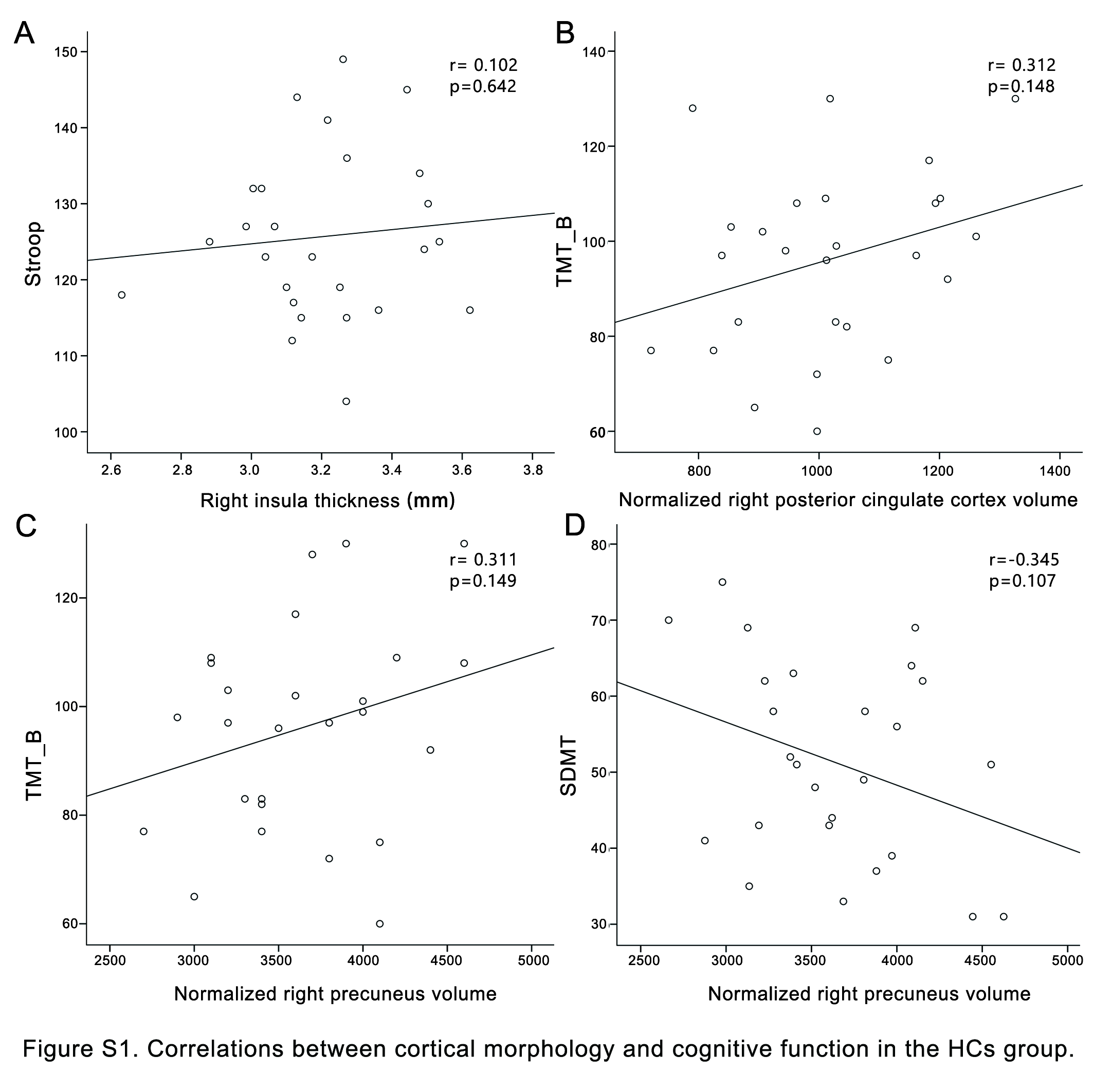

Supplement: Supplementary file 1 [file Image_1.TIF]

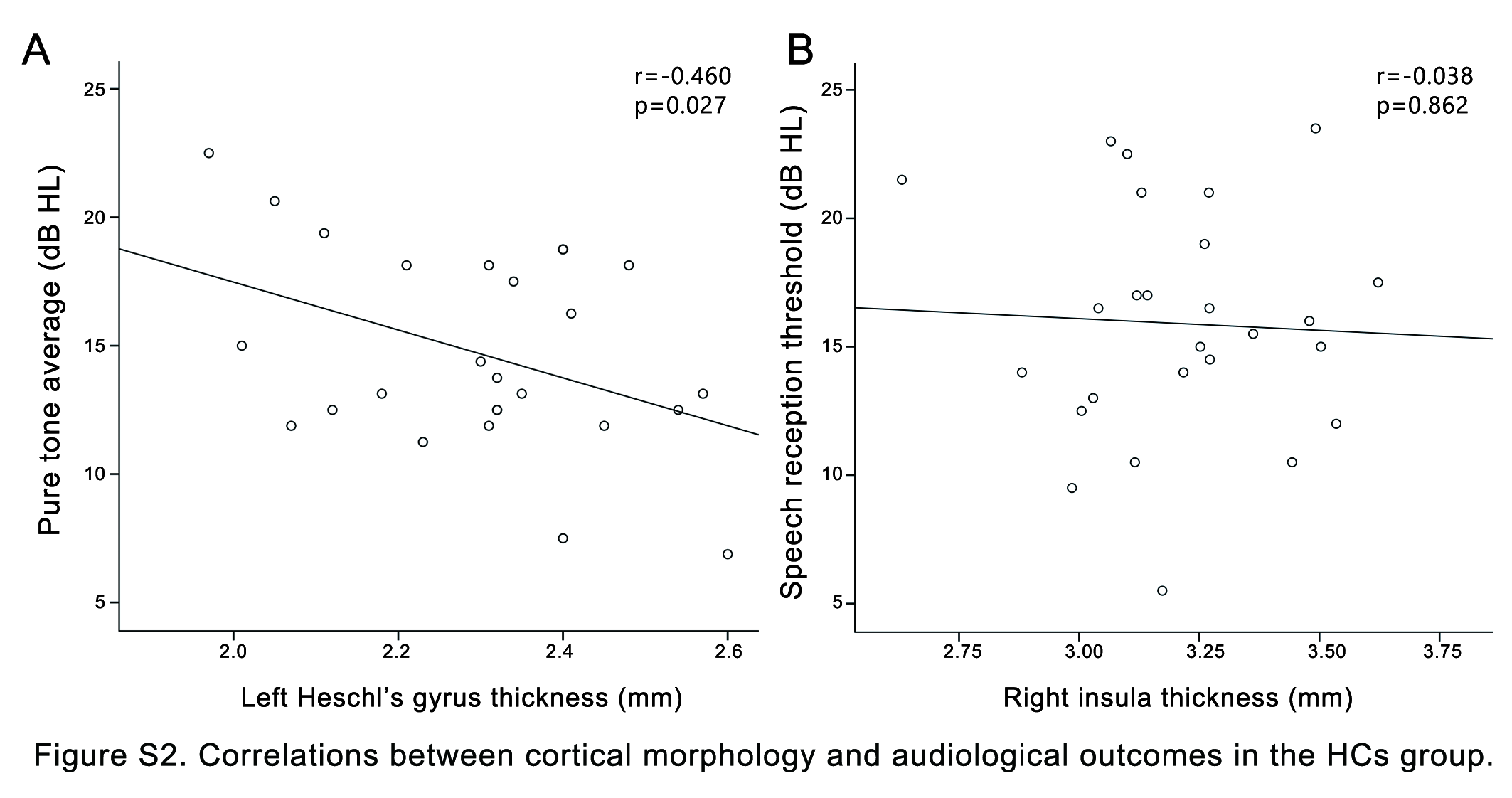

Supplement: Supplementary file 2 [file Image_2.TIF]

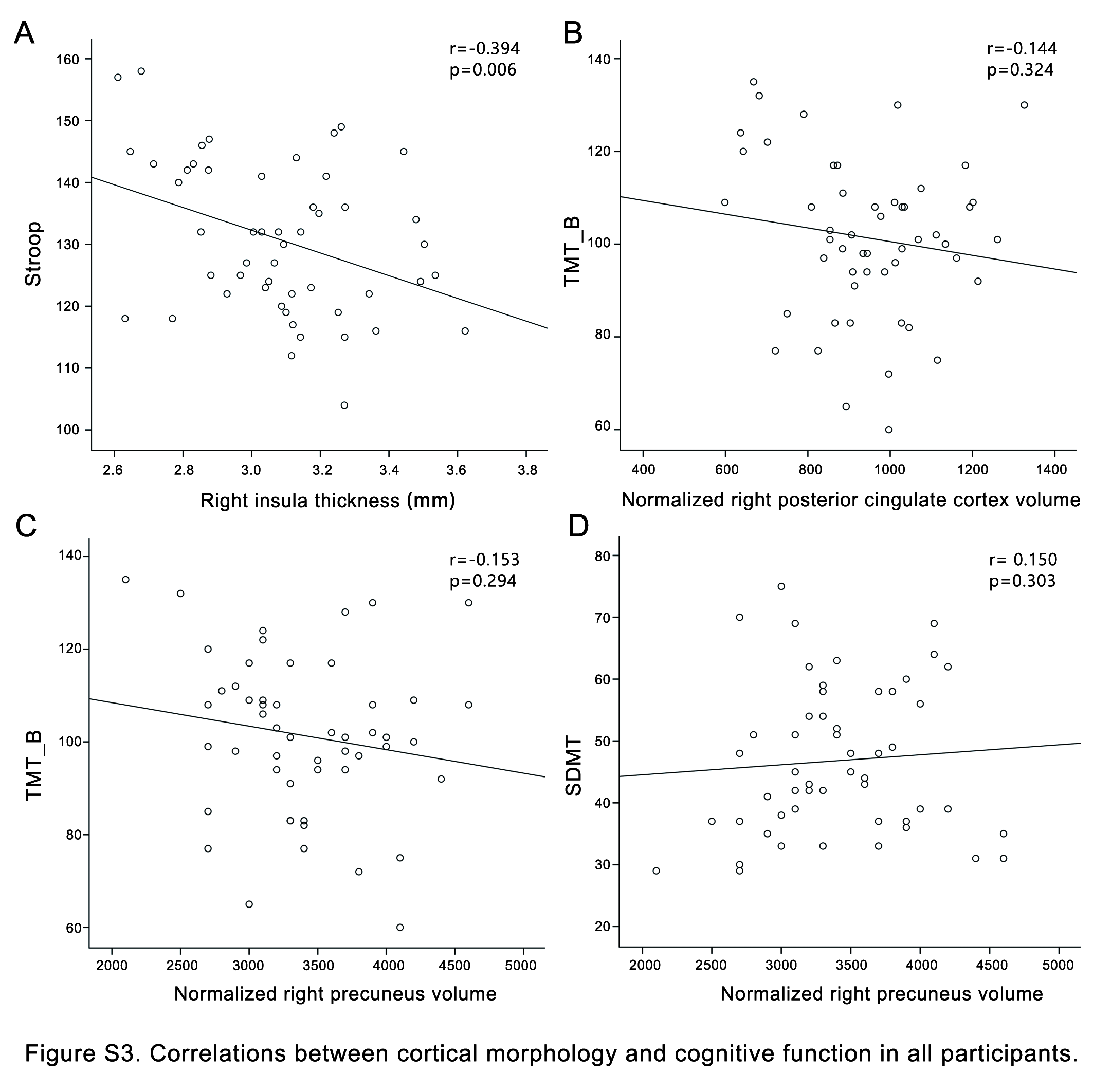

Supplement: Supplementary file 3 [file Image_3.TIF]

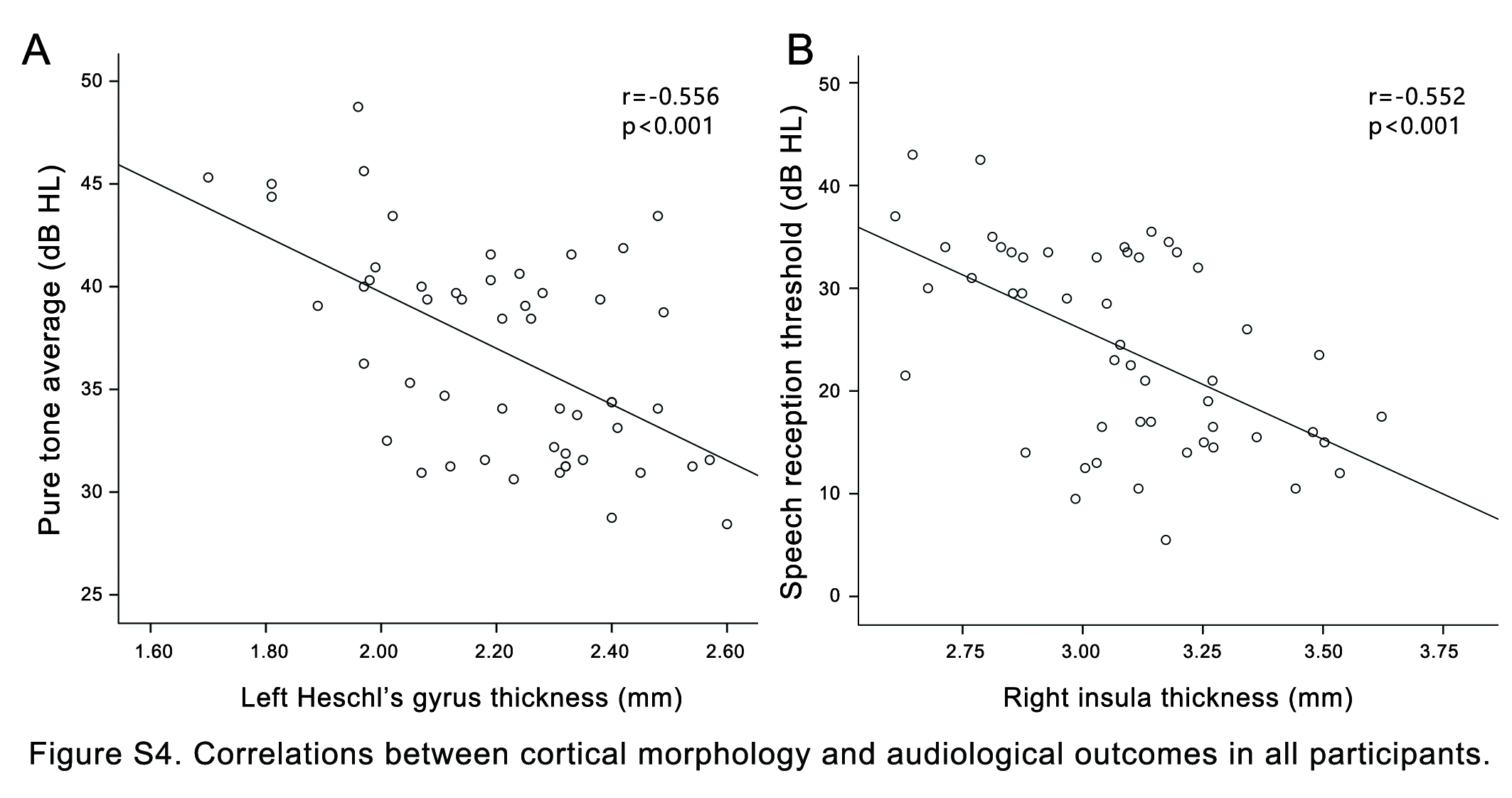

Supplement: Supplementary file 4 [file Image_4.TIF]
